# Education and practice gaps on atrial fibrillation and anticoagulation: a survey of cardiovascular nurses

**DOI:** 10.1186/s12909-015-0504-1

**Published:** 2016-01-12

**Authors:** Caleb Ferguson, Sally C. Inglis, Phillip J. Newton, Sandy Middleton, Peter S. Macdonald, Patricia M. Davidson

**Affiliations:** Faculty of Health, University of Technology Sydney, Sydney, Australia; Centre for Cardiovascular and Chronic Care, Faculty of Health, University of Technology Sydney, Sydney, Australia; St Vincent’s Health Australia (Sydney), Sydney, Australia; Australian Catholic University, Sydney, Australia; Heart Transplant Program, Sydney and Victor Chang Cardiac Research Institute, St Vincent’s Hospital, Sydney, Australia; School of Nursing, Johns Hopkins University, Baltimore, USA

**Keywords:** Clinical practice, Anticoagulant knowledge, Anticoagulation, Atrial fibrillation, Education, Survey methods, Patient education, Self-management

## Abstract

**Background:**

Patients’ knowledge of their atrial fibrillation (AF) and anticoagulation therapy are determinants of the efficacy of thromboprophylaxis. Nurses may be well placed to provide counselling and education to patients on all aspects of anticoagulation, including self-management. It is important that nurses are well informed to provide optimal education to patients. Current practice and knowledge of cardiovascular nurses on AF and anticoagulation in the Australian and New Zealand (ANZ) context is not well reported.

This study aimed to; 1) Explore the nurse’s role in clinical decision making in anticoagulation in the setting of AF; 2) Describe perceived barriers and enablers to anticoagulation in AF; 3) Investigate practice patterns in the management of anticoagulation in the ANZ setting; 4) Assess cardiovascular nurses’ knowledge of anticoagulation.

**Methods:**

A paper-based survey on current practices and knowledge of AF and anticoagulation was distributed during the Australian Cardiovascular Nursing College (ACNC) Annual Scientific Meeting, February 2014. This survey was also emailed to Cardiovascular Trials Nurses throughout New South Wales, Australia and nursing members of the Cardiac Society of Australia and New Zealand (CSANZ).

**Results:**

There were 41/73 (56 %) respondents to the paper-based survey. A further 14 surveys were completed online via nurse members of the CSANZ, and via an investigator developed NSW cardiovascular trials nurse email distribution list. A total of 55 surveys were completed and included in analyses. Prior education levels on AF, stroke risk, anticoagulation and health behaviour modification were mixed. The CHA_2_DS_2_VASc and HAS-BLED risk stratification tools were reported to be underused by this group of clinicians. Reported key barriers to anticoagulation included; fears of patients falling, fears of poor adherence to medication taking and routine monitoring. Patient self-monitoring and self-management were reported as underutilised. ANZ cardiovascular nurses reported their key role to be counselling and advising patients on therapy regimens. Anticoagulant-drug interaction knowledge was generally poor.

**Conclusion:**

This study identified poor knowledge and practice in the areas of AF and anticoagulation. There is scope for improvement for cardiovascular nurses in ANZ in relation to AF and anticoagulation knowledge and practice.

## Background

Atrial fibrillation (AF) is the most common cardiac rhythm irregularity and increases stroke risk three to five-fold [1]. The population prevalence is estimated at 2.3–3.4 %. The lifetime risk of AF is approximately 1 in 4 [1]. This incidence increases with age and rises to affect 11 % of the population over 80 years [2]. This primarily cardiogeriatric condition is characterised by chaotic electrical activity in the upper chambers of the heart. Stroke and thromboembolism are major complications of AF. Evidence-based interventions to reduce stroke risk in AF include anticoagulation and the insertion of a left atrial appendage (LAA) device, as well as the rhythm control methods such as cardioversion and catheter ablation [3]. Oral anticoagulation is the most common way to reduce stroke risk in individuals with AF. Whilst the recent advent of new oral anticoagulants (NOACs) such as the thrombin inhibitor dabigatran, and factor Xa inhibitors rivaroxaban and apixaban have held the promise of simplified dosing without the need for frequent blood testing, concerns persist of bleeding risk and the lack of any reversal agent for these agents [3]. To date, warfarin remains the most commonly used oral anticoagulant, and its use is inherently burdensome. This burden is often related to the need for routine monitoring, adaptation of lifestyle habits and more often than not; complex dosing requirements [4]. The quality of overall anticoagulation quality can be assessed by an individual’s time in therapeutic range. An individual with AF should aim to maintain their INR between 2 and 3 [5, 6]. However, many individuals may find this challenging to achieve due to a variety of factors. Unstable INRs are likely to be a consequence of medication, food or lifestyle interactions [7]. Quality anticoagulation is reliant on patients and caregivers’ knowledge of their chronic condition and anticoagulation therapy [8]. The often lifetime duration of treatment increases the complex issues of adherence, and emphasises the need for education refreshment throughout all care settings. Previous studies have highlighted that clinicians including physicians, pharmacists, dieticians and nurses fail to meet adequate knowledge levels to provide accurate and up-to-date information to patients [9, 10]. A recent European study drew attention to the need for improvement in cardiovascular nurses’ knowledge and practice on oral anticoagulant therapy [9]. With an increasing prevalence of AF, there is greater need for innovative strategies to improve patient outcomes. Strategies may include patient self-testing and self-management. The success of these strategies are dependent upon appropriate patient selection [11]. Patients must have good manual dexterity and cognitive abilities [12]. Further, they must be adept in managing the device; interpreting, understanding and taking action based on results. Self-testing and self-management should not be discounted by clinicians, as a method to improve knowledge, self-efficacy, overall quality of anticoagulation, and patient outcomes [11]. Nurses account for greater than 50 % of the health workforce in Australia and often spend the most time with patients providing clinical care. They are experienced in counselling, therefore, may be well-placed to provide patient education on anticoagulation and promote self-management during regular healthcare interactions.

## Study aim

The aim of this study was to:

1. Explore the nurse’s role in clinical decision-making in anticoagulation in the setting of AF.
2. Describe perceived barriers and enablers to anticoagulation in AF.
3. Investigate practice patterns in the management of anticoagulation.
4. Assess cardiovascular nurses’ knowledge of anticoagulation.

## Methods

### Design, setting and sample

Data were collected by survey methods. The survey was conducted at the 8th Annual Scientific Meeting of the Australian Cardiovascular Nursing College, Gold Coast, Australia on 22nd Feb 2014. All delegates attending on this date were invited to participate in the study. Additional responses were sought via an email distribution list of the Nurses Council of the Cardiac Society of Australia and New Zealand, and a state-wide cardiovascular research nurse email distribution list. Additionally, nurses working in a single site CCU of a metropolitan teaching hospital were invited to participate. *(Refer to* Table 1*for sampling frame response).*

**Table 1:** Final sampling frame response

Distribution method	Distribution sample	Number of responses	Response rate
ACNC Conference	73	41	56 %
CSANZ CNC Email list serve	320	2	0.625 %
HF Coordinator Email distribution list	31	9	29 %
Cardiology Ward	34	3	9 %
Total	458	55	12 %

*Legend*: *ACNC* Australian Cardiovascular Nursing College, *CSANZ CNC* Cardiac Society of Australia and New Zealand Cardiovascular Nursing Council, *HF* Heart Failure

### Measurements and item generation

A self-report questionnaire, recently used during a European study of cardiovascular nurses was used to assess variables related to warfarin-drug, warfarin-food interactions and knowledge on novel anticoagulants and self-management [9]. Permission to reproduce these survey items was approved by the corresponding author. The original questionnaire was reported as demonstrating good face validity. Study investigators reviewed the original survey used in Europe, and felt that the variables and items included were applicable to the ANZ setting, given the similarities in westernised healthcare systems and availability of pharmacological agents at the time of the survey. Additional investigator developed questions were included; these were developed following consultation with expert cardiovascular nurses and an extensive review of the literature. Some questions were adapted from other surveys that addressed barriers to anticoagulation or elicited the cardiovascular nurse’s role in decision-making around anticoagulation therapy [13]. The questionnaire was distributed in English.

### Data collection

All delegates were provided with a paper-based survey on their chair during the session and invited by the convenor to participate. Attendees were asked to place the completed questionnaires in a designated collection box following the session. Subsequent to the conference, the same survey was electronically distributed to nursing members of the Cardiac Society of Australia and New Zealand and via a state-wide cardiovascular research nurse email distribution list. Additionally, nurses working in the hospital CCU were invited to participate. The hospital site was selected for inclusion, due to the locality of the site in relation to the research team’s clinical practice setting. Online survey methods have previously been used to gain understanding of practice patterns in the ANZ setting [14]. Therefore, the research team believed this would yield worthy response and enhance findings from the paper-based survey. To address the potential for selection bias, a poster and box was placed in the ward setting for clinicians to provide responses. A notification for completion of the electronic survey was sent electronically once, and no reminders were sent. Within the electronic survey information, participants were advised that this survey was previously undertaken at the recent ACNC conference, and asked not to complete if they had already participated.

### Data management and data analysis

The online survey was conducted using the web-based SurveyMonkey™ platform (*www.surveymonkey.com*) and remained open until 12/05/2014. Paper based surveys were manually entered into the electronic system; this assisted in accounting for missing values. The survey ascertained the basic demographic and education characteristics of nurses related to highest qualification and length of time working in cardiovascular nursing. Descriptive analyses were used to describe the sample and the responses to study variables.

### Ethical considerations

This study was approved by the St Vincent’s Hospital, Sydney (Ref: LNR/12/SVH/62) and University of Technology Sydney (Ref: 2013000181) human research ethics committees. Approval was provided for both online and paper-based surveys at the conference. The executive committee of the Australian Cardiovascular Nursing College approved survey distribution during the conference event. All conference attendees were informed of the aims of the study. Informed consent was implied by the completion of the survey. No identifying information was collected from the participants to assure confidentiality.

## Results

### Sample characteristics, demographic information and experience

A total of 55 responses were included in the analyses. The table above identifies most responses were from the ACNC conference, with a response rate of 56 %. Unfortunately, e-survey distributed by CSANZCNC once received two responses, notwithstanding the reach of the email to 320 members. A possible reason for this may be that some of the members of CSANZCNC are also members of ACNC, and had attended the conference, and previously participated in the survey. Most respondents were female 86 % *(n = 47),* the majority of respondents 91 % *(n = 50)* came from the three most populated states in Australia (New South Wales, Victoria and Queensland). The remaining respondents were from New Zealand 9 % *(n = 5).* The majority worked in a metro area 71 % *(n = 39),* and held a Bachelor degree qualification or above 85 % *(n = 47).* 33 % (*n* = 18) identified their current role as Registered Nurse (RN). Though, the seniority of the clinicians was also reflected by the number in expert positions, 36 % *(n = 36)* identified as Clinical Nurse Consultants or Nurse Practitioners. Three respondents were not clinicians and were employed in the higher education & research sectors. Most 77 % (*n* = 40) had over 10 years of clinical experience, and 62 % *(n = 34)* had worked in cardiovascular nursing specialty practice for more than 10 years. Baseline demographic information is summarised in Table 2.

**Table 2:** Characteristics of cardiovascular nurses (*n* = 55)

Demographic variable	*n* (%)
Female *(n = 55)*	47 (86)
Highest level of education *(n = 55)*	
• Nursing training • Bachelor degree • Graduate certificate • Master’s degree • PhD • Other	5 (9) 15 (27) 11 (20) 16 (29) 5 (9) 3 (6)
Work location *(n = 55)*	
• Metro • Regional or rural	39 (70) 16 (29)
Country of workplace *(n = 55)*	
• New South Wales • Australian Capital Territory • Victoria • Western Australia • Northern Territory • Queensland • Tasmania • South Australia • New Zealand	24 (44) 0 4 (7) 0 0 22 (40) 0 0 5 (9)
Area of specialty practice *(n = 55)*	
• Chronic Heart Failure • Acute Coronary Syndrome • Coronary Care Unit • Cardiac Rehabilitation • Cardiac Step Down • Cath Lab • Research • Education • General Medicine • Other	21 (38) 8 (15) 20 (36) 8 (15) 5 (9) 5 (9) 3 (6) 3 (6) 3 (6) 1 (2)
Current position *(n = 55)*	
• Registered Nurse • Clinical Nurse Specialist • Clinical Nurse Educator • Clinical Nurse Consultant • Nurse Practitioner • Other	18 (33) 12 (22) 3 (6) 10 (18) 9 (18) 3 (6)
Years working in clinical practice *(n = 52)*	
• Less than 3 • 4–5 • 6–10 • More than 10	2 (4) 7 (8) 6 (12) 40 (77)
Years working in cardiovascular nursing *(n = 55)*	
• Less than 3 • 4–5 • 6–10 • More than 10	6 (11) 2 (4) 13 (24) 34 (62)
Years working in current department *(n = 55)*	
• Less than 3 • 4–5 • 6–10 • More than 10	13 (24) 13 (24) 14 (26) 15 (27)
Proportion of patients seen aged 65+ *(n = 54)*	
• Less than 25 % • 26–50 % • 51–75 % • 76 % or more	0 (0) 4 (7) 31 (57) 19 (35)

Participation in formal educational programs was reported to be 41–61 % across four associated topics. 48 % had attended a previous education program about AF, 41 % about stroke risk, 57 % about anticoagulation and 61 % about health behaviour modification. Previous education participation is summarised in Table 3.

**Table 3:** Previous participation in educational programs

Education program	Yes (%)
Atrial fibrillation	26 (48)
Stroke risk	20 (41)
Anticoagulation	29 (57)
Health behaviour modification	31 (61)

### Adverse outcomes

Fifty percent of respondents had cared for a patient with AF who had experienced an intracranial haemorrhage when receiving anticoagulation. And 74 % of respondents had cared for a patient with AF who had experienced a stroke whilst not receiving anticoagulation. Results are summarised in Table 4.

**Table 4:** Clinical Factors

Variable (Clinical Factor)	n (%)
Estimated prevalence of AF in CHF	
• Less than 25 % • 25–50 % • 51–75 % • 76 % or more	3 (6) 29 (54) 19 (35) 3 (6)
Have cared for a patient with AF who has experienced an ICH whilst receiving anticoagulation	27 (50 %)
Have cared for a patient with AF who has experienced a stroke whilst not receiving anticoagulation	39 (74 %)
Actively involved in MDT discussions when making treatment decisions on anticoagulation management with patients with AF	25 (46 %)

### Nurses role in decision making and anticoagulation

Results of the nurses’ role in decision making are summarised in Table 5. More than half of respondents (*54 %, n = 29*) said they were not involved in multidisciplinary team (MDT) discussions when making treatment decisions on anticoagulation management with patients with AF. Nearly half of respondents agreed (*44 %, n = 24*) or strongly agreed (*7 %, n = 4)* that the risk of stroke versus the risk of bleeding was clearly articulated to patients at commencement of anticoagulation for stroke prevention in AF. Most disagreed (*41 %, n = 22)* or strongly disagreed (*13 %, n = 7)* that they were unsure to advocate for thromboprophylaxis or not, when involved in team decisions. Most disagreed *(50 %, n = 27)* or strongly disagreed (*6 %, n = 3)* that it was difficult to decide where the benefits of thromboprophylaxis outweighed the risks of haemorrhage. Over half disagreed *(57 %, n = 30)* that they did not feel they knew enough about the risks and benefits of different anticoagulants.

**Table 5:** Clinical decision making in anticoagulation

Statement	SD	D	N	A	SA	Rating Count
	*n (%)*	*n (%)*	*n (%)*	*n (%)*	*n (%)*	
The risk of stroke versus the risk of bleeding is clearly articulated to patients when commencing anticoagulation for stroke prevention in AF	3 (6)	9 (17)	14 (26)	24 (44)	4 (7)	54
I am unsure whether to advocate for thromboprophylaxis or not when involved in team decisions	7 (13)	22 (41)	14 (26)	10 (19)	1 (2)	54
It’s difficult to decide where the benefits of thromboprophylaxis outweigh the risks of hemorrhage	3 (7)	27 (50)	10 (19)	14 (26)	0 (0)	54
I feel I do not know enough about the risk and benefits of different anticoagulants	5 (9)	30 (57)	4 (8)	12 (23)	2 (4)	53
I take time to understand my patients views on the risks and benefits of anticoagulation	0 (0)	7 (13)	10 (19)	31 (58)	6 (11)	54
Generally, my patients are well informed about the risks and benefits of anticoagulation at time of commencement	3 (6)	8 (15)	11 (20)	28 (52)	4 (7)	54
My patients receive comprehensive education about anticoagulation prior to discharge	2 (4)	5 (9)	10 (19)	26 (48)	11 (20)	54
I use the CHADS2 or CHA2DS2-VASc scores with patients to help risk stratify stroke risk in clinical practice	6 (12)	12 (23)	15 (29)	13 (25)	6 (12)	52
I use the HAS-BLED score with patients to help risk stratify bleeding risk in clinical practice	11 (22)	13 (26)	16 (31)	9 (18)	2 (4)	51
I use shared decision making with patients to explain the risks and benefits of anticoagulation for stroke prevention in AF	5 (9)	7 (13)	14 (26)	23 (43)	5 (9)	54

Most respondents *(57 %, n = 31)* agreed that they took time to understand their patients views on the risks and benefits of anticoagulation, and that they *(52 %, n = 28)* felt that generally, their patients were well informed about their risks and benefits of anticoagulation at the time of commencement. Just under half *(n = 26, 48 %)* agreed that their patients received comprehensive education about anticoagulation prior to discharge after hospitalisation. Stroke and bleeding risk stratification tools were reported to be underutilised by this group of clinicians. Specifically, only 25 % *(n = 13)* agreed that they used a CHADS_2_ or CHA_2_DS_2_-VASc tools, whilst only 18 % *(n = 9)* agreed they used the HAS-BLED tool, despite the fact that both are recommended in international guidelines for anticoagulation in AF [5]. Over half *(n = 28, 52 %)* agreed, or strongly agreed that they made use of a shared decision-making model of care to explain the risks and benefits of anticoagulation for stroke prevention in AF.

### Barriers to anticoagulation

Barriers to anticoagulation are summarised in Table 6. Barriers to anticoagulation included fears of the patient falling *(75 %, n = 39)*, fears of poor adherence to: routine monitoring *(75 %, n = 39) and* medication taking *(71 %, n = 36).* Other barriers included lack of social support (e.g. patient living alone, or a lack of a caregiver), *(41 %, n = 21)*, and fears of poor literacy *(26 %, n = 13).* Factors facilitating optimal management of thromboprophylaxis were identified, these are summarised in Tables 7 and 8.

**Table 6:** Barriers to anticoagulation

Variable (*Barrier*)	n = Y (%)	Rate count
Fear of the patient falling	39 (70)	52
Lack of social support	21 (41)	51
(e.g. patient living alone or lack of caregiver)		
Fear of poor adherence to medication taking	36 (71)	51
Fear of poor compliance to routine monitoring	39 (75)	52
(e.g. INR checking)		
Fears of poor literacy	13 (26)	50

**Table 7:** Factors facilitating optimal management of thromboprophylaxis

• Case Management • Good GP/GP support • Ease of access to INR checking • Education • Counselling assessment • Discharge planning • Follow up • Family support & involvement • A HAS-BLED and CHADS2 Score • Open discussion and understanding of thromboprophylaxis • Good understanding of risks and benefits • Pharmacy education • Regular adherence and INR monitoring	• Multi-disciplinary care • Good communication (including listening, interpreters, written info) • Warfarin booklets (written information) • Self-management & community support • Careful assessment, reassessment of factors if change • Monitoring of adherence • Ensuring type of lifestyle and therapy is matched with patients capacity to self-manage • Nurse-led anticoagulation clinics

**Table 8:** Nurse’s general comments to thromoprophyalxis use

General comments about thromboprophylaxis use in patients with AF
*“new agent dabigatran easier to manage patients”*
*“If medical officers have had a patient with a bleed, I find it makes them more cautious with subsequent patients”*
*“Some INR levels are very hard to control despite well-educated and compliant patient. Need new ideas* i.e. *Watchman LAA devices”*
*“It’s variable and physician choice”*
*“Until an antidote is developed for the newer anticoagulants we don’t use them. In some rare cases we do but often last choice”*
*“Dr’s do all the decision making. Uses CHADS*_*2*_ */ CHA*_*2*_ *DS*_*2*_ *VASc”*
*“Quick uptake of NOAC agents but often without evidence (*e.g. *prosthetic valves & AF)”*

### Practice patterns

Results of practice patterns are summarised in Tables 9 and 10. Warfarin remains the most widespread anticoagulant used in daily practice for this cohort of clinicians (100 %, *n* = 51). Less extensive use of the novel agents was reported. Only 16 % *(n = 8)* of respondents stated that most patients would be given a choice of anticoagulant, 82 % *(n = 41),* stated that ‘some’ patients may be offered new anticoagulants. 59 % *(n = 29)* of respondents stated that patients may modify therapy based on difficulties maintaining INR within therapeutic range (i.e. 2–3). Patient self-testing and self-management were not offered by 60 %, *(n = 30)* of respondents and 28 %, *(n = 14)* had never heard of such services. One respondent highlighted a possible rationale for this could be the limited remuneration and funds for the use of point-of-care machines. It was also noted by another that pharmacists had a key role in counselling and education prior to discharge following a hospitalisation. A number of respondents highlighted that the role of decision-making around anticoagulation is usually lead by the physician, cardiologist or GP and often the nurses and patients input were minimal.

**Table 9:** Cardiovascular nurses’ practice patterns; use of oral anticoagulants, involvement in oral anticoagulation therapy and use of patient INR self-management

Question	*n* (%)
Oral anticoagulants in use in daily practice	
• Warfarin (Coumadin) • Dabigatran (Pradaxa) • Rivaroxaban (Xareltro) • Apixaban (Eiliquis)	51 (100) 33 (65) 33 (65) 11 (22)
Will patients be offered a choice of drug?	
• No, all patients will be put on warfarin first • Some patients may be offered new anticoagulation • Most patients will be given a choice	1 (2) 41 (82) 8 (16)
Will patients on warfarin be able to change to one of the new drugs?	
• No, they will need to stay on warfarin • May change if difficulties keeping INR in therapeutic range • Any patient can change to one of the new drugs	1 (1) 29 (59) 19 (39)
Do you offer patients INR self-testing or self-management?	
• Both self-testing and self-management of INR • Only self-testing of INR • Neither self-testing nor self-management • Never heard about self-testing of self-management of INR	6 (12) 0 (0) 30 (60) 14 (28)
The role of nurses regarding anticoagulants in your country	
• Do not have a specific role • Counsel patients regarding adherence to drug regimen • Advice patients on dosing warfarin based on INR results • Teach about drugs, how to take them and side effects	11 (21) 34 (65) 8 (15) 33 (64)

*INR* international normalized ratio

**Table 10:** Cardiovascular nurses’ knowledge on warfarin-drug interactions

Question	*n* (%)	*n* (%)	*n* (%)	*n* (%)	
	Enhance	Inhibit	No effect	Don’t know	Rating Count
How to these anti-inflammatory agents affect oral warfarin anticoagulant therapy?					
• Aspirin • Ibuprofen • Topical salicylates	**34 (69)** 23 (48) **11 (23)**	2 (4) 8 (17) 3 (6)	9 (18) **9 (18)** 11 (23)	4 (8) 8 (17) 23 (48)	49 48 48
How do these cardiac agents affect oral warfarin anticoagulant therapy?					
• Propanolol • Cholestyramine • Atenolol	**7 (15)** 0 (0) 5 (11)	2 (4) **10 (21)** 1 (2)	15 (32) 6 (13) **20 (44)**	23 (49) 32 (67) 20 (44)	47 48 46
How do these gastrointestinal agents affect warfarin anticoagulant therapy?					
• Antiacids • Cimetidine • Metamucol • Sucralfate	0 (0) **4 (9)** 1 (2) 0 (0)	23 (49) 11 (24) 10 (22) **10 (22)**	**7 (15)** 4 (9) **5 (11)** 5 (11)	17 (36) 27 (59) 30 (65) 30 (67)	47 46 46 45
How do these vitamin supplement (s) affect oral anticoagulant therapy?					
• Multivitamin • Multivitamin and minerals • Antioxidant formula • 1200 IU vitamin E • 1000 mg vitamin C	2 (4) 2 (4) 7 (16) **18 (39)** **4 (9)**	9 (20) 9 (20) 3 (7) 5 (11) 3 (7)	**15 (33)** **15 (33)** **9 (21)** 3 (7) 12 (27)	19 (42) 19 (42) 25 (57) 20 (44) 26 (58)	45 45 44 46 45
Most antibiotics affect warfarin therapy by the process of:					
• Potentiation • Inhibition • Both • Neither (other process)		**7 (23)** 5 (17) 6 (19) 1 (4)	0 (0) **0 (0)** **1 (4)** **2 (8)**	24 (78) 25 (84) 25 (78) 23 (89)	31 30 32 26

Correct answers are shown in bold

### Cardiovascular nurses’ knowledge on warfarin interactions

The majority of respondents *(n = 34, 69 %)* answered correctly that aspirin enhanced the effect of oral warfarin anticoagulation therapy. It is of concern that 48 % *(n = 23)* of respondents answered incorrectly to interactions related to ibuprofen and 48 % *(n = 23)* did not know of interactions with topical salicylates. Whilst ibuprofen has no effect on oral anticoagulant therapy, it may impact on overall haemostasis and may increase the risk of gastrointestinal bleeding when used in combination with oral anticoagulants [15]. Only 23 % *(n = 11)* of respondents answered correctly that topical salicylates enhance oral warfarin anticoagulant therapy. Whilst results of how cardiac agents including propanolol, cholestyramine and atenolol trend towards the correct answers (propanolol enhancing, cholestyramine inhibiting and atenolol having no effect), the rate of respondents who did not know the interactions of these cardiac agents on warfarin therapy was 44–67 %. Only 9–22 % answered the questions on warfarin interactions with gastrointestinal agents correctly. More than two-thirds answered “don’t know” to the question on sucralfate-warfarin interaction. The majority of respondents did not know how any of the vitamin supplements listed affected warfarin therapy (range 42–58 % across the 5 supplements). The questions on the interaction between antibiotics and warfarin were poorly completed; the majority of respondents answered “don’t know”. Results on warfarin interactions are summarised in Table 11.

**Table 11:** Cardiovascular nurses knowledge on how to advise patients on warfarin

Question	n (%)
While on warfarin the patient:	
• Should not eat spinach • Can eat spinach once a month • Can eat as much spinach as he likes whenever he likes • Can eat spinach but needs to eat the same amount every week • *Don’t know* • *Skipped question*	9 (18) 1 (2) 2 (4) **34 (69)** 3 (6) 6
While out with friends for dinner, your patient has just finished his third glass of wine. This amount of alcohol consumed in a single evening will:	
• Cause a decrease in INR • Cause an increase in INR • Does not affect warfarin in any way • Make the patient sick when taking warfarin medication • *Don’t know* • *Skipped question*	7 (15) **26 (55)** 3 (6) 1 (2) 10 (21) 8
The best time of day to take warfarin is:	
• At lunchtime • In the evening • In the morning before breakfast • Any time of the day when you remember • *Don’t know* • *Skipped question*	0 (0) **44 (88)** 2 (4) 3 (6) 1 (2) 5
Once the patient has reached a stable warfarin dose, a PT/INR blood test:	
• Should be checked once a year • Should be checked once every 3 months • Should be checked at least once every 4 weeks • Does not need to be checked once on a stable warfarin does • *Don’t know* • *Skipped question*	0 (0) 5 (10) **45 (90)** 0 (0) 0 (0) 5
A patient just remembered that he forgot to take his warfarin medication dose last night. He/She should:	
• Skip in the dose of warfarin he/she missed • Take the missed warfarin dose right now • Wait and take two doses right now • Take one-half of the missed dose of warfarin right now • *Don’t know* • *Skipped question*	**29 (58)** 8 (16) 0 (0) 8 (16) 5 (10) 5
Once the patient’s warfarin is stopped, how long does it take to get the medication out of his/ her system?	
• 5 h • 5 days • 5 weeks • 5 months • *Don’t know* • *Skipped question*	1 (2) **38 (76)** 2 (4) 0 (0) 9 (18) 5
After starting warfarin, how long (in months/years) would you expect the patient to be taking warfarin?	
• 1 year • 1 month • It depends on each person’s needs • If you start warfarin you will have to be on the medication for the rest of your life • *Don’t know* • *Skipped question*	0 (0) 0 (0) **43 (84)** 9 (18) 0 (0) 4
Women who are pregnant:	
• Should not take warfarin • Can safely take warfarin during the second and third trimester • Can take warfarin but only need to take it every others day • Would not need to take warfarin, since being pregnant prevents them from getting blood clots • *Don’t know* • *Skipped question*	24 (48) **6 (12)** 0 (0) 0 (0) 20 (40) 5

Correct answers are shown in bold

### Cardiovascular nurses’ knowledge on warfarin related advice

There were eight questions that assessed cardiovascular nurse’s knowledge on warfarin advice. Responses are summarised in Table 11. Most questions were answered correctly with the exception of advice around warfarin use and pregnancy. 70 % *(n = 34)* of respondents knew that patients who are taking warfarin can consume spinach, however that they need to eat the same amount regularly every week. Over half *(55 %, n = 26)* correctly answered that consuming three glasses of wine will cause an increased in INR. Yet, 21 % *(n = 10)* of respondents did not know the answer to this question. 88 % *(n = 44)* of cardiovascular nurses knew that the best time of day to take warfarin was the evening, and 90 % *(n = 45)* were aware that patients with a stable INR should have it checked every 4 weeks. Only 58 % *(n = 29)* of nurses would have given correct advice to patients on the action to take if a patient remembered missing a last dose. Over three quarters *(76 %, n = 38)* of respondents correctly answered that once warfarin is ceased, it takes 5 days to be cleared from the patient’s body. Most *(84 %, n = 43)* nurses correctly identified that the length of time a patient is expected to be taking warfarin is patient centric, and dependant on individual needs. Only 12 % *(n = 6)* of respondents correctly answered that women who are pregnant can safely take warfarin during the second and third trimester. Less than half, 48 % *(n = 24)* of respondents incorrectly answered this question, wrongly identifying that pregnant women should not take warfarin, which is concerning around the correct information provision to this patient population.

## Discussion

This study demonstrates that cardiovascular nurses in Australia and New Zealand have insufficient knowledge on oral anticoagulant therapy, warfarin-diet, and warfarin-medication interactions. Our findings are consistent with recent international research [7, 9]. The less extensive reported use of novel agents may be due to their gradual introduction to the Pharmaceutical Benefit Scheme (PBS). The PBS is the national pharmaceutical rebate scheme providing medicines at a government-subsidised price. It is of concern that such a small percentage stated that most patients would be given a choice of anticoagulant. There remains considerable scope for improvement in this area of shared decision-making.

The lack of knowledge of warfarin-medication interactions is alarming. It is of concern at the lack of knowledge on warfarin related advice, particularly pertaining to pregnancy and how alcohol affects INR. Our findings represent a typically older and more experienced cardiovascular nursing population, working in specialised positions with advanced qualifications. And as such, are likely to be more knowledgeable on anticoagulation than other nurses. Given the overall poor results, it is feared that knowledge is likely to be even poorer in the broader nursing population.

### Practice patterns

The cardiovascular nurses surveyed stated that warfarin remains the most widespread oral anticoagulant for stroke prevention in AF, whilst NOACs are reported to have lesser usage. It is concerning that only 16 % of respondents stated that most patients would be given a choice. It appears that there is scope for improvement in the practice of shared decision-making and patient-centred care. It is important that nurses maintain an active role in the decision-making processes and act as an advocate for patients and caregivers. This may be particularly problematic when patients are presented with complex risk calculators and benefit statistics of various treatments. Patient self-testing and self-management strategies have been not yet been embraced as innovative practices that support able patients. Barriers to patient self-management and self-testing include frailty, poor manual dexterity and cognitive dysfunction [12]; however these practices should not be discounted by clinicians. Additionally, a poor rebate for self-testing devices may also impede uptake in Australia and New Zealand. The nurse’s role in education and counselling on anticoagulation was highlighted. Some respondents expressed that they often delegate this to the pharmacist for education. This practice appears commonplace, and is worrisome. A respondent expressed *“most times, little education is given”.* This has immense implication for the quality of thromboprophylaxis.

### Knowledge

Cardiovascular nurses must ensure that they are up-to-date with current evidence-based information on AF and anticoagulation therapy to inform the education and care they provide to patients. Incorrect and inaccurate knowledge on drug-drug, drug-food interactions and monitoring requirements was prevalent among the respondents to our survey. This may lead to inappropriate patient counselling and education. This would adversely impact patient outcomes. It is therefore vital that cardiovascular nurses are knowledgeable and keep abreast with new information related to AF and anticoagulation practices. Support for self-testing and self-management practices must be preferred over teaching alone. Nurses are best-placed to provide ongoing counselling throughout the spectrum of care; from hospitalisation to discharge and within primary care settings. However, the quality of this counselling is dependent on a strong contemporary knowledge base. Cardiovascular nurses must refrain from simple task delegation of anticoagulation education to pharmacists or other clinicians, it is important to adopt a combined and comprehensive approach. This may assist in optimising therapy, improving issues of adherence and ultimately patient outcomes. Clinicians must engage further with this topic to ensure safe care. Results from this study highlight the need for cardiovascular nurses to maintain a contemporary knowledge base. The Australian Health Practitioner Regulation Agency (AHPRA) mandates that all registered nurses must participate in at least 20 h of continuing nursing professional development per year that is relevant to the context of practice [16]. Education modules specific to AF and anticoagulation, including stroke and bleeding risk and lifestyle modification may be of assistance in maintaining minimum standards for continuing professional development for cardiovascular nurses.

## Limitations

This study has some limitations. Firstly, whilst the response rate of 58 % to the paper-based survey distributed during the conference is encouraging, the generalizability of these findings is difficult. These findings represent a typically older and more experienced cardiovascular nursing population, working in specialised positions with higher qualifications. Therefore this may not be representative of the general Australian and New Zealand cardiovascular nursing population whom provide bedside patient education on anticoagulation. Secondly, the majority of respondents (80 %) were attending a cardiovascular nursing conference, for many of the workforce this remains a privilege to secure funding and time to attend such professional development events. The seniority of respondents may reflect the demographic that normally attends conferences, hold a professional society membership or has access to a workplace email account. Again, this limits the generalizability of findings.

## Conclusions

The results of our study demonstrate that of the cardiovascular nurses surveyed in Australia and New Zealand most had inadequate knowledge on oral anticoagulant therapy. There is need for improvement, to ensure quality of care for patients with AF receiving anticoagulation. Cardiovascular nurses need to be able to provide accurate, robust and timely advice to patients to topics including lifestyle, medication and food interactions to anticoagulation. A lack of knowledge on these topics may contribute to inappropriate counselling and education. Further, the communication of inaccurate information may implicitly reinforce myths and misconceptions around anticoagulation. The education of patients on anticoagulation is not a role of a single health professional. A team approach must be taken and nurses have a key role in providing answers to questions and sound clinical advice across all care settings. Future research should address modes of delivery of AF and anticoagulation education for clinicians, individuals and their caregivers.

**Table:** 

Implications for Clinical Practice
• ANZ cardiovascular nurses need to improve their knowledge on oral anticoagulant therapy.
• Lack of clinician knowledge may lead to inaccurate patient advice and impact adherence to therapy.
• Including a comprehensive education program pre-discharge may help to improve the quality and safety of anticoagulation
• Cardiovascular nurses need to be more actively involved in anticoagulation decision making in the clinical setting.
• Due to the duration of therapy for this chronic condition, there is need for education refreshment and re-assessment of patients and clinicians knowledge, across all care settings.
• There is need to explore the scope for professional organisations to credential nurses on AF and anticoagulation.

## References

[1] Ball J, Carrington M, McMurray JJV, Stewart S (2013). Atrial fibrillation: Profile and burden of an evolving epidemic in the 21st century. Int J Cardiol.

[2] Shea JB, Sears SF (2008). A patient’s guide to living with atrial fibrillation. Circulation.

[3] Ferguson C, Inglis SC, Newton PJ, Middleton S, Macdonald PS, Davidson PM. Atrial fibrillation: Stroke prevention in focus. *Australian Critical Care.* 2013;27(2):92–98. 10.1016/j.aucc.2013.08.002 24054541

[4] Ferguson C, Inglis SC, Newton PJ, Middleton S, Macdonald PS, Davidson PM (2013). Atrial fibrillation and thromboprophylaxis in heart failure: The need for patient-centered approaches to address adherence. Vasc Health Risk Manag.

[5] Camm AJ, Lip GY, De Caterina R, Savelieva I, Atar D, Hohnloser SH (2012). 2012 focused update of the ESC Guidelines for the management of atrial fibrillation. Eur Heart J.

[6] Wann LS, Curtis AB, January CT, Ellenbogen KA, Lowe JE, Estes M (2011). 2011 ACCF/ AHA/ HRS focused update on the management of patients with atrial fibrillation: a report of the american college of cardiology foundation/ american heart association task force on practice guidelines. Circulation.

[7] Couris R, Tataronis G, McCloskey W, Oertel L, Dallal G, Dwyer J (2006). Dietary vitamin K variability affects International Normalized Ratio (INR) coagulation indices. *International journal for vitamin and nutrition research. Internationale Zeitschrift fur Vitamin- und Ernahrungsforschung*. Int J Vitam Nutr Res.

[8] Ferguson C, Inglis SC, Newton PJ, Middleton S, Macdonald PS, Davidson PM (2015). The caregiver role in thromboprophylaxis management in atrial fibrillation: A literature review. Eur J Cardiovasc Nurs.

[9] Oterhals K, Deaton C, De Geest S, Jaarsma T, Lenzen M, Moons P (2014). European cardiac nurses’ current practice and knowledge on anticoagulation therapy. Eur J Cardiovasc Nurs.

[10] Couris RR, Tataronis GR, Dallal GE, Blumberg JB, Dwyer JT (2000). Assessment of healthcare professionals’ knowledge about warfarin-vitamin K drug-nutrient interactions. J Am Coll Nutr.

[11] Heneghan C, Alonso-Coello P, Garcia-Alamino JM, Perera R, Meats E, Glasziou P (2006). Self-monitoring of oral anticoagulation: a systematic review and meta-analysis. Lancet.

[12] Dolor RJ, Ruybalid RL, Uyeda L, RG Edson, Phibbs C, Vertees JE (2010). An evaluation of patient self-testing competency of prothrombin time for managing anticoagulation: pre-randomization results of VA Cooperative Study #481--The Home INR Study (THINRS). J Thromb Thrombolysis.

[13] Gattellari M, Worthington J, Zwar N, Middleton S (2008). Barriers to the use of anticoagulation for nonvalvular atrial fibrillation. Stroke.

[14] Newton P, Davidson P, Sanderson C (2012). On behalf of the improving palliative care through clinical trials group. An online survey of Australian physicians reported practice with the off-label use of nebulised frusemide. BMC Palliat Care.

[15] Ansell J, Hirsh J, Hylek E, Jacobson A, Crowther M, Palareti G (2008). Pharmacology and management of the vitamin K antagonists. Chest.

[16] Australian Health Practitioner Regulation Agency (AHPRA). Nursing and Midwifery Continuing Professional Development Registration Standard. 2010. Accessed 4th August 2014, 2014.

